# Effects of the Local Bone Renin-Angiotensin System on Titanium-Particle-Induced Periprosthetic Osteolysis

**DOI:** 10.3389/fphar.2021.684375

**Published:** 2021-06-24

**Authors:** Zhiping Zhao, Changyao Wang, Yingxing Xu, Xiangyu Wang, Bin Jia, Tengbo Yu, Yingzhen Wang, Yongtao Zhang

**Affiliations:** ^1^ Department of Orthopedics, The Affiliated Hospital of Qingdao University, Qingdao, China; ^2^ Medical Department of Qingdao University, Qingdao, China

**Keywords:** periprosthetic osteolysis, renin-angiotensin system, titanium particles, perindopri, RANKL and wnt/β-catenin signaling pathways

## Abstract

Wear particles may induce osteoclast formation and osteoblast inhibition that lead to periprosthetic osteolysis (PPOL) and subsequent aseptic loosening, which is the primary reason for total joint arthroplasty failure. Local bone renin-angiotensin system (RAS) has been found to participate in the pathogenic process of various bone-related diseases via promoting bone resorption and inhibiting bone formation. However, it remains unclear whether and how local bone RAS participates in wear-particle-induced PPOL. In this study, we investigated the potential role of RAS in titanium (Ti) particle-induced osteolysis *in vivo* and osteoclast and osteoblast differentiation *in vitro*. We found that the expressions of AT1R, AT2R and ACE in the interface membrane from patients with PPOL and in calvarial tissues from a murine model of Ti-particle-induced osteolysis were up-regulated, but the increase of ACE in the calvarial tissues was abrogated by perindopril. Moreover, perindopril mitigated the Ti-particle-induced osteolysis in the murine model by suppressing bone resorption and increasing bone formation. We also observed in RAW264.7 macrophages that Ang II promoted but perindopril suppressed Ti-particle-induced osteoclastogenesis, osteoclast-mediated bone resorption and expression of osteoclast-related genes. Meanwhile, Ang II enhanced but perindopril repressed Ti-particle-induced suppression of osteogenic differentiation and expression of osteoblast-specific genes in mouse bone marrow mesenchymal stem cells (BMSCs). In addition, local bone RAS promoted Ti-particle-induced osteolysis by increasing bone resorption and decreasing bone formation through modulating the RANKL/RANK and Wnt/β-catenin pathways. Taken together, we suggest that inhibition of RAS may be a potential approach to the treatment of wear-particle-induced PPOL.

## Introduction

Total joint arthroplasty (TJA) is widely used for the treatment of end-stage joint diseases, which could significantly relieve pain in patients and improve joint function (Learmonth et al., 2007). However, aseptic loosening, initiated by periprosthetic osteolysis (PPOL) induced by wear particles, is the primary reason for prosthesis failure (Wang Q. et al., 2020). Increasing evidence indicates that wear particles released from the surface of prostheses, such as titanium (Ti) particles, promote bone resorption by inducing excessive activation of osteoclast, leading to PPOL and subsequent aseptic loosening (Hu et al., 2017; Liao et al., 2021). Furthermore, recent studies have shown that Ti particles induce PPOL by inhibiting bone formation (Wang et al., 2016; Wang L. et al., 2020). Nevertheless, the precise mechanism underlying the pathogenesis of wear-particle-induced PPOL has not been elucidated, and effective prophylactic therapies for this morbidity are not available.

The renin-angiotensin system (RAS), a circulating endocrine system, has an important role in regulating blood pressure and electrolyte homeostasis. It also plays a crucial part in regulating the remodeling in various organs and tissues, including the bone tissues and synovial tissues (Zhao et al., 2019; Zhang et al., 2020). In both systemic and local RAS, angiotensin II (Ang II) is a major biologically active hormone, which is converted from angiotensin I (Ang I) by angiotensin converting enzyme (ACE). It functions through angiotensin type 1 and type 2 receptors (AT1R and AT2R). Studies have shown that the main components of RAS, including ACE, AT1R, and AT2R, are expressed in human and animal bone and synovial tissues, and participate in many osteolytic diseases such as rheumatoid arthritis, avascular necrosis of the femoral head, and osteoporosis through modulating bone resorption and bone formation (Zhang et al., 2014; Akagi et al., 2020; Mo et al., 2020). Wang et al. (2018) demonstrated that the RAS components were expressed and up-regulated in synovial tissues of rheumatoid arthritis patients and collagen-induced arthritis rats, and that aberrant activation of RAS in synovial tissues participated in the pathogenesis of rheumatoid arthritis by increasing bone resorption and decreasing bone formation. Zhou et al. (2017) found that bone RAS increased bone resorption and decreased bone formation by promoting osteoclast differentiation and repressing osteoblast formation, which eventually resulted in osteopenia in ovariectomized mice. Zhang et al. (2014) revealed that activation of local bone RAS could be a significant reason for steroid-induced osteonecrosis in rabbits. In line with this, our previous work identified that local bone RAS inhibited bone formation and stimulated bone resorption in glucocorticoid-induced osteoporosis (Yongtao et al., 2014). Of note, Zhang et al. (2011) found that a low-dose of captopril decreased the production of osteolytic inflammatory cytokines by down-regulating the expressions of RAS components, thereby inhibiting Ti-particles-induced inflammatory osteolysis which was a potential mechanism of the pathogenesis of PPOL. However, little is known about how local bone RAS participates in regulating the process of wear-particle-induced bone destruction and osteogenic inhibition, as well as subsequent PPOL.

Receptor activator of nuclear factor-κB ligand (RANKL) is an important factor for the formation and activation of osteoclasts. Binding of RANKL to its receptor, RANK, activates nuclear factor (NF)-κB by recruiting adaptor molecules, including tumor necrosis factor receptor-associated factor 6 (TRAF6). This in turn leads to the activation of downstream signaling transcriptional factors, such as C-FOS and nuclear factor of activated T-cells 1 (NFATC1), which up-regulates the expression of osteoclast-related genes and promotes the maturation and activation of osteoclast (Gohda et al., 2005; Park et al., 2017). Osteoprotegrin (OPG), a competitive antagonist of RANKL, can block the combination of RANKL and RANK, thereby inhibiting the formation and activity of osteoclast (Tsukasaki et al., 2020). The RANKL-RANK signaling pathway is a crucial one among the many signaling pathways that regulate osteoclast formation and activation. It has been proved to promote osteoclastogenesis in wear-particle-induced PPOL (Wu et al., 2015; Zhu et al., 2017; Wei et al., 2020). Moreover, the effect of local bone RAS on osteoclast has been reported to be closely related to the RANKL-RANK signaling pathway (Shimizu et al., 2012; Wang et al., 2018). For example, Shimizu et al. (2008) demonstrated that local bone RAS activated the RANKL-RANK signaling pathway and promoted osteoclast formation and activation when RANKL interfered with osteoclast precursor cells. Tanaka et al. (2018) indicated that bone RAS induced the expressions of RANKL and osteoclast genes, and promoted osteoclast formation and bone resorption.

The Wnt/β-catenin signaling pathway is crucial for osteogenic differentiation and bone formation (Molagoda et al., 2019; Chu et al., 2020; Zhou et al., 2020). Recent studies have demonstrated that local RAS suppresses bone formation and osteogenic differentiation through modulating the Wnt/β-catenin pathway (Tanida et al., 2013; Klavdianou et al., 2017). In our previous works, we observed that Ang II, the main component of RAS, inhibited osteogenic differentiation of isolated fibroblast-like synovial cells by increasing DKK-1, a potent inhibitor of the canonical Wnt/β-catenin signaling (Zhang et al., 2020), and activation of RAS in the synovium promoted periarticular osteopenia by increasing bone resorption and decreasing bone formation through regulating the RANKL/RANK/TRAF6 and Wnt/β-catenin signaling pathways (Wang et al., 2018). Moreover, studies on wear-particle-induced PPOL suggest that the inhibition of bone formation induced by wear particles is regulated by the Wnt/β-catenin signaling pathway (Wang et al., 2016; Ping et al., 2017; Wang et al., 2021).

In spite of the extensive existing studies, it remains unclear whether and how local bone RAS participates in wear-particle-induced PPOL and the potential mechanism in this pathogenesis. In the present study, we investigated whether local bone RAS participated in Ti-particle-induced osteoclastogenesis of RAW264.7 macrophages, suppression of osteogenesis of mouse bone marrow mesenchymal stem cells (BMSCs), and mouse calvarial osteolysis. We further investigated the potential roles of the RANKL/RANKL and Wnt/β-catenin signaling pathways in Ti-particle-induced osteolysis.

## Materials and Methods

### Patients and Samples

The present study was approved by the Ethical Committee of the Affiliated Hospital of Qingdao University, China. All the patients agreed to donate interface membrane or synovium, and provided with written informed consent. Interface membrane samples were obtained from four patients with PPOL who underwent revised total hip arthoplasty (one female and three males; age 65–78 years). Synovial tissue samples were obtained from four patients with femoral neck fracture who underwent total hip arthoplasty (one female and three males; age 60–72 years). Interface membrane and synovial tissue samples were used separately in three independent experiments for each of the assays described below (*n* = 3 per sample). Part of each tissue sample was fixed in 4% paraformaldehyde and embedded in paraffin for hematoxylin and eosin (H&E) staining and immunohistochemistry. The remaining tissues were frozen and stored at −80°C for Western blot and reverse-transcription quantitative polymerase chain reaction (RT-qPCR) analysis.

### Preparation of Ti Particles

Commercial pure Ti particles with an average diameter of 4 μm were purchased from Alfa Aesa (Catalog #00681, Tewksbury, United States). To eliminate endotoxin, the particles were baked for 6 h at 180°C and washed in 75% ethanol for 48 h before suspension and storage in sterile phosphate-buffered saline (PBS) at 4°C (Ren et al., 2019). After that, the particle suspension was sterilized using a high-pressure sterilizer and only particles free of endotoxin were used in the subsequent experiments.

### Titanium-Particle-Induced Mouse Calvarial Osteolysis Model

To determine the effect of bone local RAS on Ti-particle-induced osteolysis *in vivo*, a mouse calvarial osteolysis model was established as previously described (Zhai et al., 2014; Shao et al., 2015). Briefly, 30 healthy 8 week-old male C57BL/6J mice were randomly assigned to three groups (*n* = 10): sham PBS control (sham group), Ti particles with PBS (Ti group), and Ti particles with perindopril (Ti + Peri group). After anesthesia, the calvarial periosteum was separated and 20 mg of pretreated Ti particles was embedded under the periosteum at the middle suture of the calvarium in the Ti group and the Ti + Peri group. In the sham group, the incision was closed without further intervention. During the next 2 weeks, mice in the Ti + Peri group were injected intraperitoneally with perindopril (Servier, France, 20 mg/kg/day), while those in the sham and Ti groups were injected with the same dose of PBS. Three mice from each group were selected randomly and injected intraperitoneally with calcein (20 mg/kg, Sigma) and alizarin red (40 mg/kg, Sigma) 10 and 3 days before sacrifice, respectively, to identify newly formed bone. Calvarial tissues were used separately in three independent experiments for each of the assays described below (*n* = 3 per group). Part of the calvarial tissues were fixed in 80% ethanol for 3 days and embedded in methyl methacrylate. Some tissues were fixed with 4% paraformaldehyde for micro-CT analysis, and were then decalcified in 10% EDTA and embedded in paraffin for histological analysis. The remaining tissues were frozen and stored at −80°C for further analysis. The animal experimental procedures complied with the Guide for the Care and Use of Laboratory Animals published by the US National Institutes of Health. The research protocols were approved by the Ethical Committee of Animal Experiments of Qingdao University.

### Micro-CT Scanning

The fixed calvarial tissues were analyzed using the micro-CT Skyscan 1276 system (Bruker, Germany). The scanning protocol was set at an isotropic voxel size of 6.53 μm, X-ray energy settings of 85 kV and 200 uA, and an exposure time of 400 ms, with a rotation in equiangular steps of 0.9° following an angle of 180°. The wear particles were removed before scanning to avoid metal artifacts. Reconstruction was accomplished by Nrecon (version 1.7.3.0). A volume of interest (VOI) of 3 mm × 3 mm × 1 mm was selected for further quantitative analysis. The bone volume to tissue volume ratio (BV/TV), bone mineral density (BMD), and total porosity of each sample were obtained using CT Analyser software (Skyscan). The experiment was performed in triplicate.

### Histological and Histomorphometric Analysis

The paraffin-embedded calvarial tissue sections (4 μm) were prepared for H&E staining and tartrate resistant acid phosphatase (TRAP) staining. The TRAP staining was performed using a commercial TRAP kit (Nanjing Jiancheng Biotechnology). The specimens were observed and photographed under a high-quality microscope. The number of TRAP-positive multinucleated osteoclasts, the ratio of osteoclast surface to bone surface (OCs/BS) and the ratio of eroded surface to bone surface (ES/BS) were analyzed. The experiment was performed in triplicate.

### Fluorescence Calcein and Alizarin Red Labeling

The methyl methacrylate-embedded undecalcified calvarial tissues were cut into 5 μm sections, and calcein/alizarin red labeling was captured using a fluorescence microscope (EVOS FL, Invitrogen by Thermo Fisher Scientific, German). Bone formation and bone mass parameters, including mineral apposition rate (MAR), bone formation rate per bone surface (BFR/BS) and ratio of mineralizing surface to bone surface (MS/BS), were evaluated. The experiment was performed in triplicate.

### Cell Culture and Treatment

Mouse RAW264.7 macrophages were purchased (ATCC, Manassas, United States) and cultured in Dulbecco’s modified Eagle’s medium (DMEM) containing 10% fetal bovine serum (FBS, Gibco, Grand Island, United States), 1% penicillin (Gibco, United States) and 1% streptomycin (Gibco, United States) at 37°C in 5% CO_2_ and 95% humidity. Mouse BMSCs used in this study were isolated and purified as previously described (Huang et al., 2015), and the experimental protocols were approved by the institutional animal care and use committee. Briefly, mice were euthanized and their hind limbs were aseptically resected immediately. The soft tissues and ends of the hind limbs were removed, and the marrow tissues of both the femur and tibia were flushed with low glucose DMEM containing 20% fetal bovine serum, 1% penicillin and streptomycin. Cells were seeded into 6 cm^2^ cell culture flasks and incubated at 37°C in 5% CO_2_ and 95% humidity. Non-adherent cells were removed when the medium was changed every 3 days, while adherent cells were cultured until 70–80% confluence and then passaged. Cells from passages 3–5 were used in subsequent experiments. RAW264.7 macrophages and mouse BMSCs were used separately in three independent experiments for each of the assays described below. In the following steps, Ang II (MedChemExpress, 10 um) or perindopril (Servier, France, 1 um) was dissolved in sterile water and added to the culture medium with or without addition of Ti particles (0.1 mg/ml). For the control groups, only sterile water was added.

### Osteoclastogenesis *In Vitro*

RAW264.7 macrophages were cultured in 96-weel plates at a density of 1 × 10^4^ cells/well with complete DMEM overnight. Then, 50 ng/ml M-CSF (R&D Systems, Minneapolis, MN, United States) and 100 ng/ml RANKL (R&D Systems, Minneapolis, MN, United States) were added into the medium, with the addition of sterile water and/or Ti particles in the presence or absence of Ang II (10 um) or perindopril (1 um). The obtained osteoclastogenic induction medium was renewed every 3 days until mature osteoclasts were observed. The experiment was performed in triplicate.

### Cell Counting Kit-8 Assay

RAW264.7 macrophages were seeded in 96-well plates (1 × 10^4^ cells/well) and treated as described above. The cells were cultured in the induction medium for 48 h. Then, CCK-8 solution (Dojindo Laboratories, China) was added into each well according to the manufacturer’s instructions. The effects of Ti particles alone or in combination with Ang II or perindopril on cell viability were measured using a microplate reader to read the optical density at an absorbance of 450 nm. The experiment was performed in triplicate.

### Tartrate Resistant Acid Phosphatase Staining

RAW264.7 macrophages were cultured in 96-weel plates (1 × 10^4^ cells/well) and treated as above. The cells were cultured in the induction medium for 7 days. After that, cells were washed three times with PBS, fixed with 4% paraformaldehyde for 20 min, and then stained using a TRAP Kit (Solarbio, China). TRAP-positive cells with more than three nuclei were identified as osteoclasts and counted using Image Pro-Plus 6.0 (Media Cybernetics, Bethesda, MD, United States). The experiment was performed in triplicate.

### Resorption Pit Assay

To examine osteoclast-mediated bone resorption, RAW264.7 macrophages were cultured into 24-well osteo assay surface plates (Corning, New York, United States) at a density of 1 × 10^5^/well and treated as described above. The cells were cultured in the induction medium for 7 days, and were then washed by sonication until cells were completely removed from the plates. An inverted microscope was used to snap the images of bone resorption pits, and Image Pro-Plus 6.0 software was used to quantify the area of bone resorption. The experiment was performed in triplicate.

### Osteogenic Differentiation *In Vitro*

Mouse BMSCs were seeded in 24-well plates at 5 × 10^4^ cell density and cultured overnight in low glucose complete DMEM. The medium was then removed and replaced with osteogenic induction medium containing 100 nm dexamethasone (Sigma, United States), 10 mm β-glycerophosphate (Sigma, United States) and 50 μm ascorbic acid (Sigma, United States). The induction medium was changed every 3 days. The experiment was performed in triplicate.

### Alkaline Phosphatase Staining

Mouse BMSCs were seeded in 24-well plates and treated as above described. After 7 days osteogenic induction, cells were fixed with 4% paraformaldehyde for 15 min and incubated with alkaline phosphatase (Beijing Solarbio Science and Technology, China) at 37°C for 20 min under shading treatment. After that, images were obtained by a microscope. The experiment was performed in triplicate.

### Alkaline Phosphatase Activity Assessment

Alkaline Phosphatase (ALP) activity was assessed by colorimetric assay. Briefly, mouse BMSCs were seeded in 24-well plates and treated with osteogenic induction medium for 7 days. Then, the cells were lysed with luciferase lysis buffer (Promega, United States) and the lysate was centrifuged at 12,000 × g for 10 min at 4°C. Following that, 5 μl sample lysate was mixed with 5 μl ALP substrate (BD Biosciences Clontech, United States) and 15 μl LUPO buffer (containing 10 mM diethanolamine, 0.5 mM MgCl2 and 10 mM L-homoarginine). The mixture was incubated at 37°C for 30 min and then ALP activity was measured by luciferase assay. The experiment was performed in triplicate.

### Alizarin Red Staining

The mineralization of mouse BMSCs was determined by alizarin red staining. Cells were seeded in 24-well plates, treated as above described, and cultured in osteogenic induction medium for 21 days. Then, the cells were rinsed with PBS, fixed with 4% paraformaldehyde for 15 min, and stained with alizarin red (Sigma, United States) at room temperature for 2 min. Images were obtained by a microscope and calcium nodules were detected as red bodies. For quantitative analysis, alizarin red was extracted using 10 % acetic acid (Sigma, United States) and the optical density was measured at 405 nm. The experiment was performed in triplicate.

### Immunohistochemistry

Sections (4 μm) of synovial tissue, interface tissue and calvarial tissue were prepared. After a regular deparaffinization procedure, the sections were incubated with 0.3% hydrogen peroxidase for 15 min at room temperature. Subsequently, antigen retrieval was performed using 0.01 M citrate buffer at 80°C for 20 min. Next, the sections were blocked with normal goat serum for 30 min at room temperature, and incubated with primary antibodies against AT1R (Abcam, United Kingdom, 1:200), AT2R (Abcam, United Kingdom, 1:200), and ACE (Abcam, United Kingdom, 1:200). Finally, the sections were incubated with biotinylated secondary antibody (Zhongshan Golabrige Biotechnology, China) followed by horseradish peroxidase (HRP)-conjugated streptavidin (Zhongshan Golabrige Biotechnology, China). The ultimate reaction product was visualized with diaminobenzidine. The stained signals were captured and photographed by the Leica Microsystems (Wetzlar, Germany). The experiment was performed in triplicate.

### Western Blot

Tissues were lysed by radioimmunoprecipitation assay (RIPA) lysis buffer (Beyotime, China) containing 1% protease inhibitor (MedChemExpress) cocktail on ice, and the tissue samples were centrifuged at 10,000 × *g* for 10 min at 4°C. The supernatants were collected to quantify the protein concentration using the bicinchoninic acid (BCA) protein assay reagent (Beyotime, China). The obtained protein samples combined with loading buffer were heated at 95°C for 5 min. Next, equivalent amounts of protein samples were loaded onto 10% SDS poly acrylamide gels, and electrophoresis was conducted for 120 min at a constant voltage of 90 V, and then protein was transferred onto polyvinylidene fluoride (PVDF) membranes. The membranes were blocked for 2 h with 5% fat-free milk dissolved in Tris-buffered saline-Tween (TBST) and then incubated overnight at 4°Cwith primary antibodies including AT1R (Abcam, United Kingdom, 1:1,000), AT2R (Abcam, United Kingdom, 1:1,000), ACE (Abcam, United Kingdom, 1:1,000), RANKL (Abcam, United Kingdom, 1:1,000), TRAF6 (Abcam, United Kingdom, 1:1,000), NFATC1 (Cell Signaling Technology, United States, 1:1,000), C-FOS (Cell Signaling Technology, United States, 1:1,000), β-catenin (Cell Signaling Technology, United States, 1:1,000), and GAPDH (Cell Signaling Technology, United States, 1:2,000), respectively. After being washed three times with Tris-buffered saline-Tween, the blots were incubated with HRP-conjugated secondary antibodies (Cell Signaling Technology, United States, 1:5,000). The target bands were visualized using ECL-PLUS reagents (Sigma, United States) and scanned using a BioSpectrum Imaging System (UVP, Thermo Fisher Scientific, United States). The results were quantified by integrated density using Image J software and were normalized to GAPDH levels. The experiment was performed in triplicate.

### Reverse-Transcription Quantitative Polymerase chain reaction

Total RNA was extracted from RAW264.7 macrophages, mouse BMSCs, and tissues by TRIzol reagent (TaKaRa, Japan) under RNase-free conditions, and RNA concentration and purity were determined using an ultraviolet spectrophotometer (Implant, Munich, Germany). After that, total RNA was reversely transcribed into cDNA using the Revert Aid First Strand cDNA Synthesis kit (TaKaRa, Japan). The obtained cDNA was diluted and used as PCR templates. The mRNA levels of target genes were detected using the SYBR Green PCR master mix (TaKaRa, Japan) and all PCR samples were run in triplicate. Real-time quantitative PCR was performed using a LightCycler® 96 system (Roche, Indianapolis, IN, United States). The reaction conditions were as follows: 95°C for 10 min; 95°C for 10 s, 60°C for 10 s, and 72°C for 15 s for forty cycles. The relative expressions of the target genes were normalized to the GAPDH levels and analyzed by the 2^−ΔΔCt^ method. The sequences of primers were designed using Primer3 Plus and are provided in Table 1. The experiment was performed in triplicate.

**TABLE 1:** Primers sequences for reverse transcription-quantitative polymerase chain reaction.

Target	Primer sequence (5′-3′)	GenBank no
AT1R(H)	F: TCCAAGATGATTGTCCCA	NM_032049.3
	R: CTATCACCACCAAGCTGT	
AT2R(H)	F: ACCAATCTGTCATCTACC	NM_000686.5
	R: CAAGCATTCACTCCTAAG	
GAPDH(H)	F: AACGGGAAGCTTGTCATC	NM_001357943.2
	R: ACTCCACGACGTACTCAG	
AT1R(M)	F: CGT​CTA​CCA​CAT​GCA​CCG​TGA​AC	NM_009642.5
	R: GCA​GCA​GCG​TCT​GAT​GAT​GAG​TC	
AT2R(M)	F: GGA​TGA​ACA​GCG​GCC​TAG​AAC​AC	NM_007429.5
	R: CGT​CTT​CGT​CCT​CGT​CCT​CCT​C	
TRAP(M)	F: CAA​GAA​CTT​GCG​ACC​ATT​GTT​A	NM_001102404.1
	R: ATC​CAT​AGT​GAA​ACC​GCA​AGT​A	
CK(M)	F: GCT​TGG​CAT​CTT​TCC​AGT​TTT​A	NM_007802.4
	R: CAA​CAC​TGC​ATG​GTT​CAC​ATT​A	
OSCAR(M)	F: GAATGCTTTGCCTGTATG	NM_175632.3
	R: CTAGAGGACCTTGGTTGG	
OCN(M)	F: CCAAGCAGGAGGGCAATA	NM_001032298.3
	R: TCGTCACAAGCAGGGTCA	
Col1a1(M)	F: GAG​CGG​AGA​GTA​CTG​GAT​CG	NM_007742.4
	R: GCT​TCT​TTT​CCT​TGG​GGT​TC	
Runx2(M)	F: CCGGTCTCCTTCCAGGAT	NM_001271630.1
	R: GGGAACTGCTGTGGCTTC	
Osterix(M)	F: TCG​TCT​GAC​TGC​CTG​CCT​AGT​G	NM_130458.4
	R: CTG​CGT​GGA​TGC​CTG​CCT​TG	
RANKL(M)	F: GGA​AGC​GTA​CCT​ACA​GAC​TAT​C	NM_011613.3
	R: AAA​GTG​GAA​TTC​AGA​ATT​GCC​C	
TRAF6(M)	F: GAA​AAT​CAA​CTG​TTT​CCC​GAC​A	NM_001303273.1
	R: ACT​TGA​TGA​TCC​TCG​AGA​TGT​C	
C-FOS(M)	F: TCT​CTA​GTG​CCA​ACT​TTA​TCC​C	NM_010234.3
	R: GAG​ATA​GCT​GCT​CTA​CTT​TGC​C	
NFATC1(M)	F: GAG​AAT​CGA​GAT​CAC​CTC​CTA​C	NM_016791.4
	R: TTG​CAG​CTA​GGA​AGT​ACG​TCT​T	
β-Catenin(M)	F: GGTCCGAGCTGCCATGTT	NM_001165902.1
	R: TGGCAAGTTCCGCGTCAT	
GAPDH(M)	F: GAG​AGA​GGC​CCA​GCT​ACT​CG	NM_008084.3
	R: GAG​GGC​TGC​AGT​CCG​TAT​TTA	

### Statistical Analysis

Statistical analyses were performed using SPSS 21.0 (SPSS Inc., Chicago, IL, United States). Data are expressed as the mean ± standard deviation (SD). Differences among groups were analyzed using Kruskal–Wallis test, one-way analysis of variance test, or Student’s t-test; subgroup analysis was performed using the LSD test. A value of *p* < 0.05 was considered statistically significant for all analyses.

## Results

### Renin-Angiotensin System Expression Was Up-Regulated in the Interface Membrane From Patients With Periprosthetic Osteolysis

**FIGURE 1:**
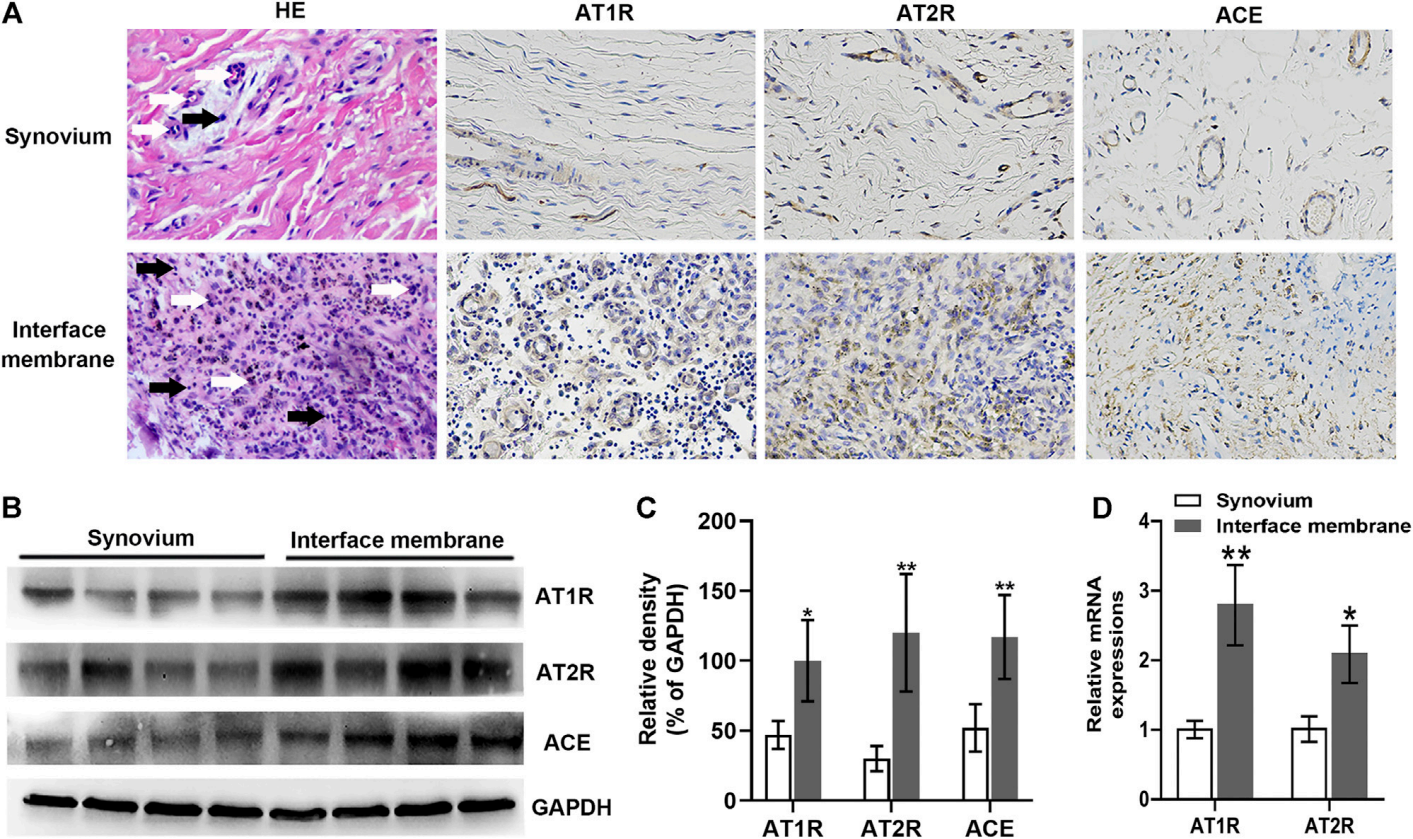
Up-regulated AT1R, AT2R, and ACE expressions in the interface membrane tissues from patients with PPOL. The levels of AT1R, AT2R, and ACE in the interface membrane tissue from patients with PPOL and the synovial tissue from patients with femoral neck fracture were determined by H and E staining, immunohistochemistry, Western blot, or RT-qPCR. Data are representative images or expressed as the mean ± SD of each group from three separate experiments (*n* = 3 per group). **(A)** Representative H and E staining and immunohistochemistry images (magnification ×400). Black arrows point at lymphocytes, and white arrows point at macrophages. **(B,C)** Western blot analyses. **(D)** RT-qPCR analyses. *p < 0.05 vs. the synovium group; **p < 0.01 vs. the synovium group.

AT1R, AT2R, and ACE expressions were characterized by immunohistochemistry. In comparison with those in the synovium from patients with femoral neck fracture, the expressions of AT1R, AT2R, and ACE in the interface membrane from patients with PPOL were up-regulated (Figure 1A). H&E staining revealed infiltration of a large number of lymphocytes and macrophages into the interface membrane (Figure 1A). Western blot showed that the relative ratios of AT1R, AT2R, and ACE to GAPDH proteins in the interface membrane were significantly increased (*p* < 0.05, Figures 1B,C). In addition, higher levels of AT1R and AT2R mRNA were detected in the interface membrane (Figure 1D). Taken together, the expression of RAS was up-regulated in the interface membrane from PPOL patients.

### Renin-Angiotensin System Expression Was Up-Regulated in Calvarial Tissues of Titanium-Particle-Induced Osteolysis

**FIGURE 2:**
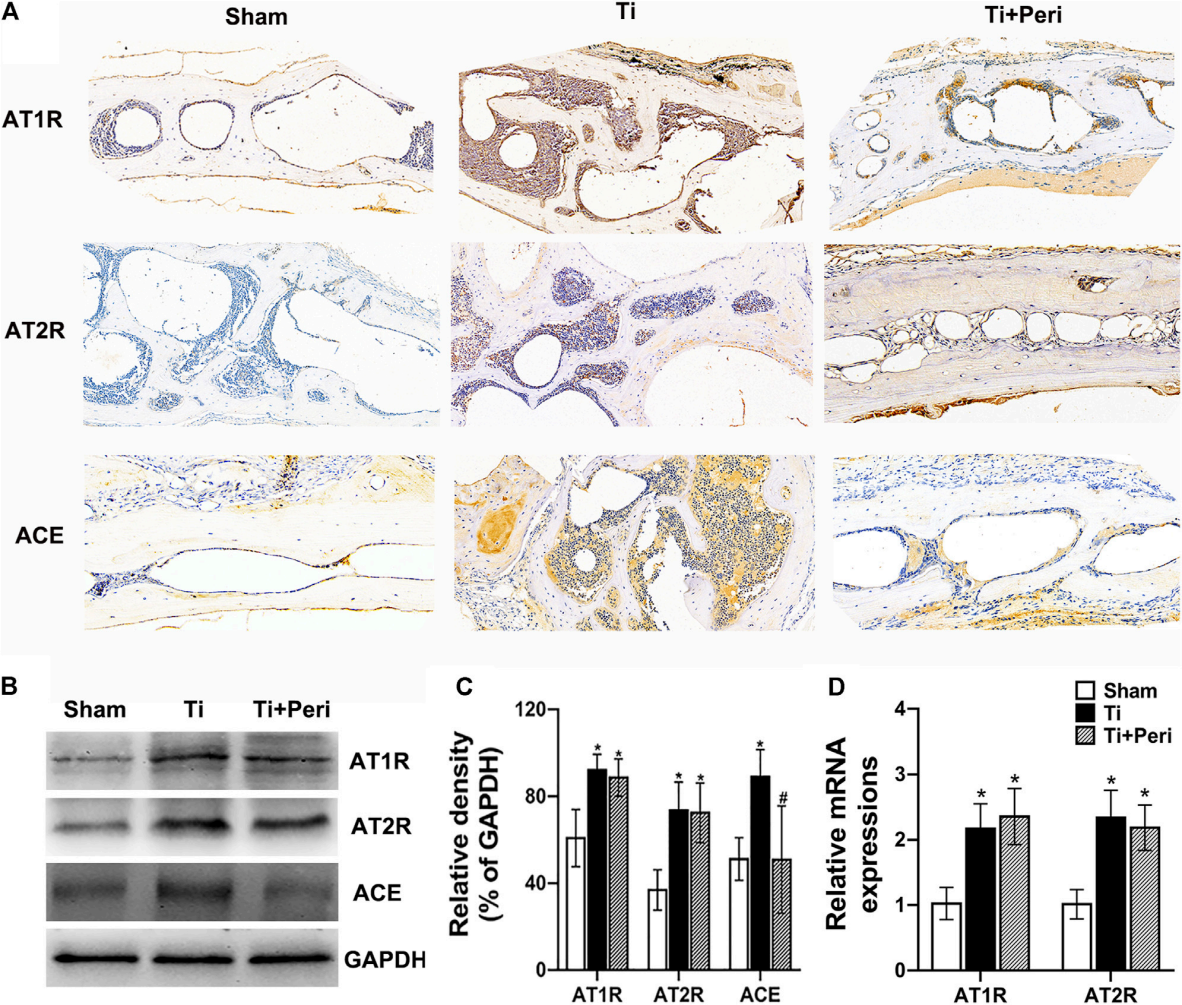
Up-regulated AT1R, AT2R, and ACE expressions in the mice calvarial tissue of Ti-particle-induced osteolysis. The levels of AT1R, AT2R, and ACE in mice calvarial tissue samples were determined by immunohistochemistry, Western blot or RT-qPCR. Data are representative images or expressed as the mean ± SD of each group from three separate experiments (*n* = 3 per group). **(A)** Representative immunohistochemistry images (magnification × 400). **(B,C)** Western blot analyses. **(D)** RT-qPCR analyses. *p < 0.05 vs. the sham group; #*p* < 0.05 vs. the Ti group.

AT1R, AT2R, and ACE expressions in the mouse calvarial tissues were examined by immunohistochemistry. The results showed that AT1R, AT2R, and ACE were also expressed in mouse calvarium, and the expressions of AT1R, AT2R, and ACE were up-regulated in the Ti group (Figure 2A). The protein levels of AT1R, AT2R, and ACE significantly increased in the Ti group but the expression of ACE remarkably decreased in the Ti + Peri group (Figures 2B,C). Furthermore, the relative mRNA levels of AT1R and AT2R significantly increased in the Ti group (Figure 2D).These results demonstrated that the expression of RAS was up-regulated in the calvarial tissues in the pathogenic process of Ti-particle-induced osteolysis.

### Inhibition of ACE Mitigated Titanium-Particle-Induced Osteolysis in the Murine Calvarial Osteolysis Model

**FIGURE 3:**
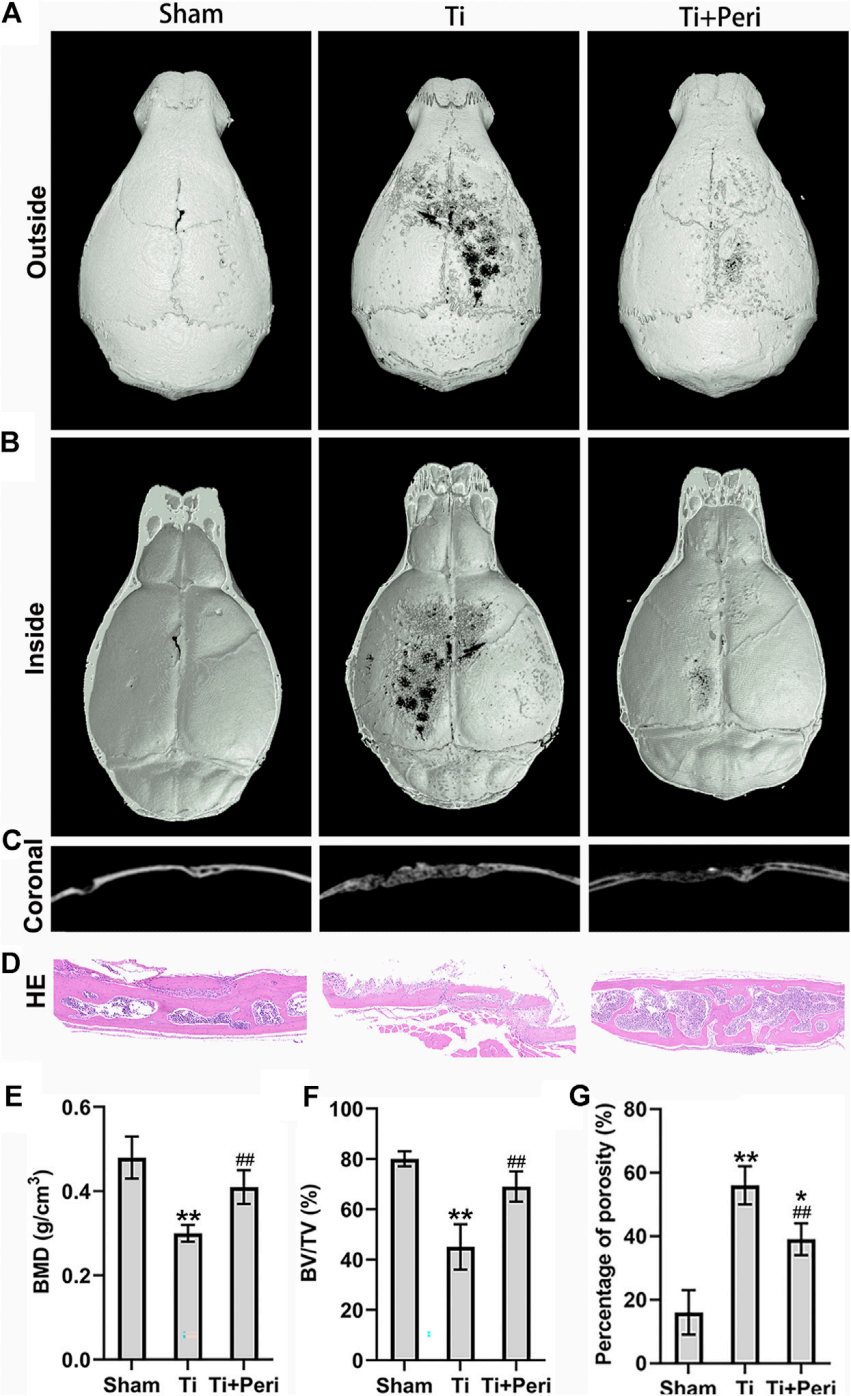
Inhibition of ACE mitigated Ti-particle-induced osteolysis in a murine calvarial model. Data are representative images or expressed as the mean ± SD of each group from three separate experiments (*n* = 3 per group). **(A–C)** Three-dimensional reconstructed images of representative micro-CT from each group. **(D)** Representative H and E staining images (magnification × 400). **(E)** Quantitative analyses of BMD (g/cm^2^). **(F)** Quantitative analyses of BV/TV (%). **(G)** The percentage of total porosity (%). *p < 0.05 vs. the sham group; **p < 0.01 vs. the sham group; ##*p* < 0.01 vs. the Ti group.

Micro-CT 3D reconstruction demonstrated a large area of bone erosion and a number of bone resorption pores on the calvarial surface in the Ti group. Whereas, such phenomena were significantly mitigated in the Ti + Peri group (Figures 3A–C). H&E staining revealed extensive erosion of the calvarial surface in the Ti group, which was remarkably reduced by the treatment with perindopril (Figure 3D). The results of quantitative analysis indicated significantly reduced BMD and BV/TV (Figure 3E,F) and increased percentage of total porosity (Figure 3G) in the Ti group as compared with in the sham group, and such effects were markedly mitigated by the treatment with perindopril. In short, inhibition of ACE alleviated Ti-particle-induced osteolysis in the mouse calvarial osteolysis model.

### Inhibition of ACE Ameliorated Titanoum-Particle-Induced Increase of Bone Resorption and Decrease of Bone Formation in the Murine Calvarial Osteolysis Model

**FIGURE 4:**
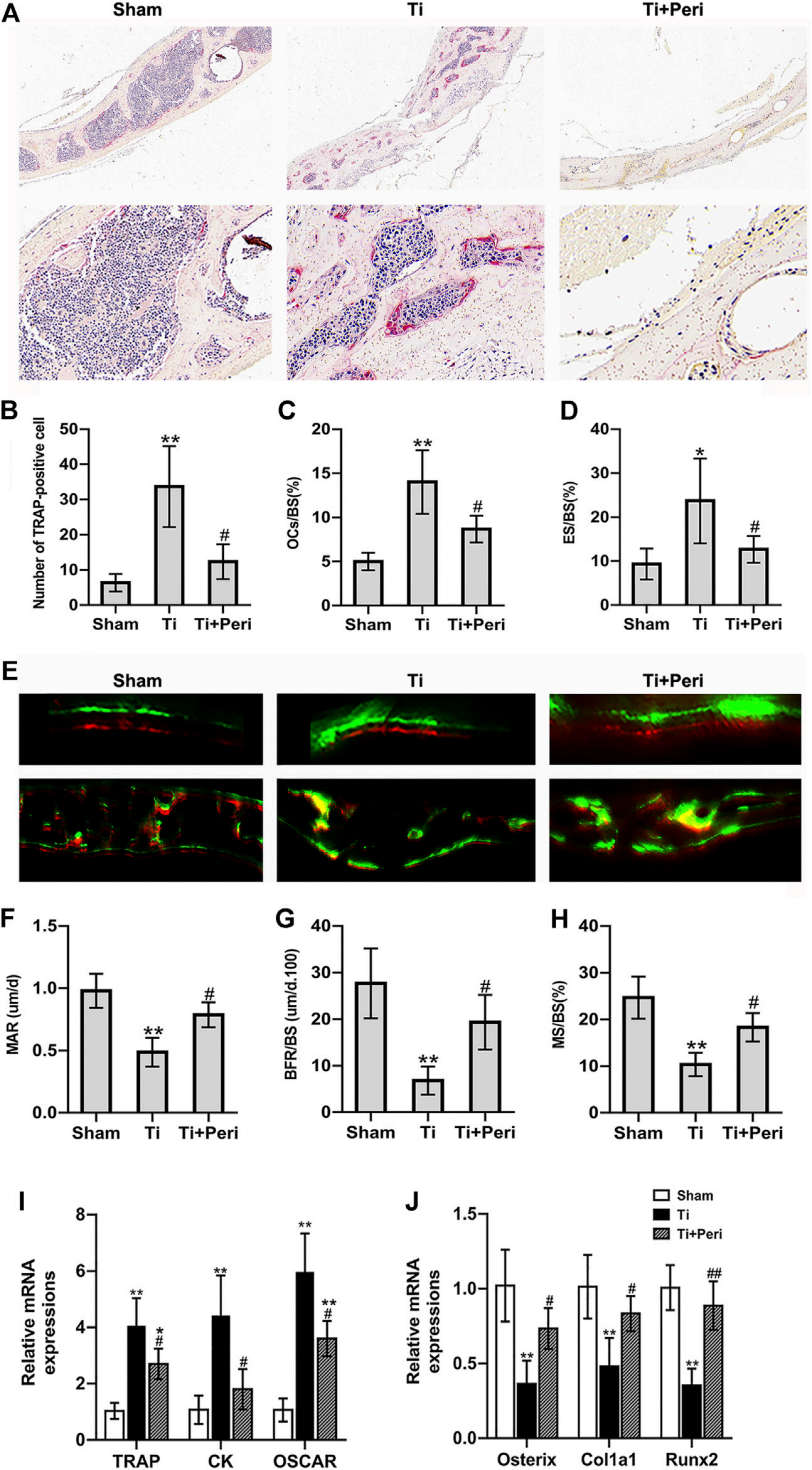
Inhibition of ACE ameliorated Ti-particle-induced increase in bone resorption and decrease in bone formation in a murine calvarial osteolysis model. Data are representative images or expressed as the mean ± SD of each group from three separate experiments (*n* = 3 per group). (A) Representative TRAP staining images (magnifications ×100 and ×400). **(B)** Quantitative analyses of the number of TRAP-positive cell. **(C)** Quantitative analyses of OC.s/BS (%). **(D)** Quantitative analyses of ES/BS (%). **(E)** Representative fluorescence calcein and alizarin red labelling images (magnifications ×100 and ×400). **(F)** Quantitative analyses of mass parameters, including mineral apposition rate (um/d). **(G)** Quantitative analyses of BFR/BS (um/d.100). **(H)** Quantitative analyses of mineralizing surface to bone surface (%). **(I)** RT-qPCR analyses of the relative levels of osteoclast-specific genes (TRAP, CK, and OSCAR) in mice calvarial tissue to the control GAPDH expression. **(J)** RT-qPCR analyses of the relative levels of osteoblast-specific genes (Osterix, Col1a1, and Runx2) in mice calvarial tissue to the control GAPDH expression. *p < 0.05 vs. the sham group; **p < 0.01 vs. the sham group; #*p* < 0.05 vs. the Ti group; ##*p* < 0.01 vs. the Ti group.

Histological staining and histomorphometric analysis confirmed the effect of perindopril on Ti-particle-induced bone resorption *in vivo*. TRAP staining revealed an increased area of bone erosion with increased number of osteoclasts on the calvarial surface in the Ti group as compared with in the other two groups (Figures 4A). Quantitative analysis showed that the number of TRAP-positive cells and the values of OCs/BS and ES/BS in the Ti group were significantly greater than those in the other two groups (Figures 4B–D). RT-qPCR indicated that perindopril significantly reduced Ti-particle-stimulated relative mRNA levels of osteoclast-specific genes, including TRAP, cathepsin K (CK) and osteoclast associated receptor (OSCAR) in calvarial tissues (Figure 4I).

To assess the effect of perindopril on bone formation, the calvarial tissue samples were stained with fluorescent alizarin red after calcein treatment, and were examined by fluorescent microscopy. As shown in Figure 4E, calcein staining and alizarin red staining fluorescent signals were obviously observed in the three groups, and the distance between the two fluorescent signals was smaller in Ti group than in other groups. And quantitative analysis exhibited that the mass parameters, including mineral apposition rate, BFR/BS and mineralizing surface to bone surface values in the Ti group were significantly lower than those in the sham group, which were significantly elevated in the Ti + Peri group (Figures 4F–H). Furthermore, RT-qPCR demonstrated that perindopril significantly increased Ti-particle-induced relative mRNA levels of osteoblast-related genes, such as osterix, collagen1a1 (Col1a1), and runt-related transcription factor 2 (Runx2) in calvaria tissues (Figure 4J). Collectively, these *in vivo* data demonstrated that inhibition of ACE ameliorated the increased bone resorption and decreased bone formation induced by Ti partciles.

### Ang II Promoted but Perindopril Suppressed Titanium-Particle-Induced Osteoclastogenesis, Osteoclast-Mediated Bone Resorption, and Expression of Osteoclast-Related Genes

**FIGURE 5:**
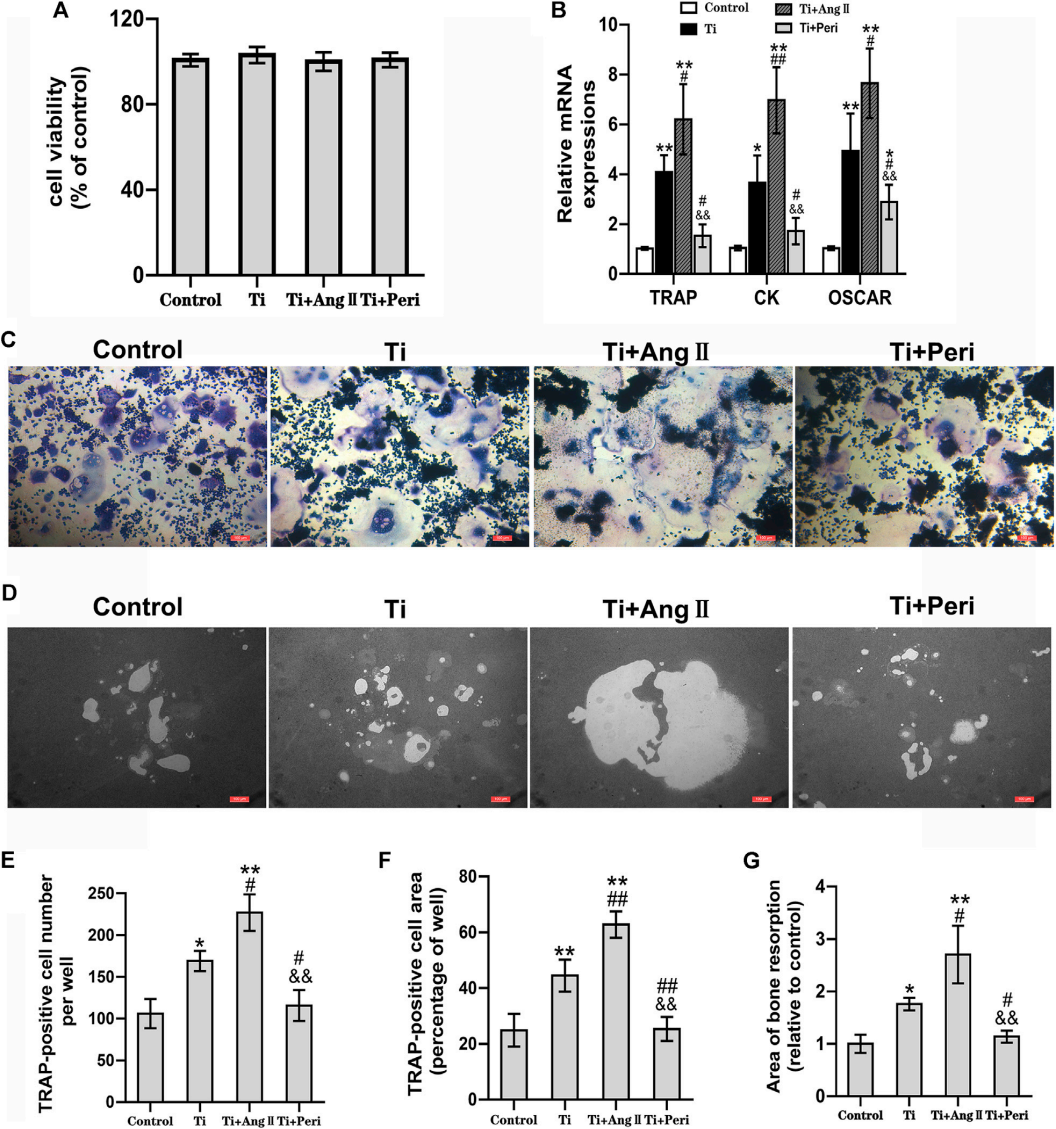
Ang II promoted but perindopril suppressed Ti-particle-induced osteoclastogenesis, osteoclast-mediated bone resorption, and expression of osteoclast-related genes in RAW264.7 macrophages. Data are representative images or expressed as the mean ± SD of each group from three separate experiments (*n* = 3 per group). **(A)** CCK8 assay of cell viability 48 h after treatment. **(B)** RT-qPCR analyses of relative levels of osteoclast-specific genes (TRAP, CK, and OSCAR) to the control GAPDH expression on day five. **(C)** TRAP staining for osteoclast formation and differentiation on day seven. Representative TRAP staining images (magnification ×100). **(D)** Representative images of bone resorption in osteo assay plates (magnification ×100) on day seven. **(E)** The number of TRAP-positive cells per well. **(F)** The area of TRAP-positive cells per well. **(G)** The area of resorption per well. *p < 0.05 vs. the control group; **p < 0.01 vs. the control group; #*p* < 0.05 vs. the Ti group; ##*p* < 0.01 vs. the Ti group; &&*p* < 0.01 vs. the Ti + Ang II group.

The effects of Ti particles alone or with Ang II or perindopril on cell viability were investigated in RAW264.7 macrophages by the CCK8 assay. The results showed no significant differences in cell viability under different treatment conditions (Figure 5A). Meanwhile, TRAP staining was performed to determine the role of Ang II or perindopril in Ti-particle-induced osteoclastogenesis. The results revealed that RAW264.7 macrophages differentiated into characteristic TRAP-positive multinucleated osteoclasts (Figure 5C), and Ti particles significantly promoted the differentiation. Furthermore, the number and area of TRAP positive osteoclasts increased substantially in the Ti + Ang II group. In cells exposed to both Ti and perindopril, the number and area of TRAP positive osteoclasts significantly decreased as compared with those in cells treated with Ti particles alone (Figures 5E,F). On the whole, these *in vitro* results indicated that Ti particles promoted osteoclastogenesis, which was enhanced by Ang II but inhibited by perindopril.

Considering that Ti-particle-induced osteoclast differentiation was obviously promoted by Ang II but remarkably reversed by perindopril, we further investigated whether Ang II or perindopril was involved in regulating Ti-particle-induced osteoclastic bone resorption. As shown in Figure 5D, a part scale of bone resorption area was observed in the control group. The area increased in the Ti group, indicating that Ti particles could induce osteoclast-mediated bone resorption. Furthermore, the area increased substantially in the Ti + Ang II group but decreased in the Ti + Peri group (Figure 5G), which suggested that Ang II enhanced but perindopril inhibited osteoclast-mediated bone resorption induced by Ti particles.

Upregulation of osteoclast-related genes plays a significant role in osteoclast differentiation. The roles of Ang II or perindopril in Ti-particle-induced expression of osteoclast-related genes were investigated by RT-qPCR. The results showed that the mRNA levels of TRAP, CK, and OSCAR genes were elevated in the Ti group as compared with in the control group. In addition, the effect of Ti particles inducing the expression of osteoclast-related genes was furhter enhanced by Ang II but remarkably reduced in the Ti + Peri group (Figure 5B). Consistent with the results of TRAP staining and bone resorption pit assay, Ang II promoted but perindopril suppressed Ti-particle-induced expression of osteoclast-related genes *in vitro*.

### Ang II Enhanced but Perindopril Repressed Titanium-Particle-Induced Suppression of Osteogenic Differentiation

**FIGURE 6:**
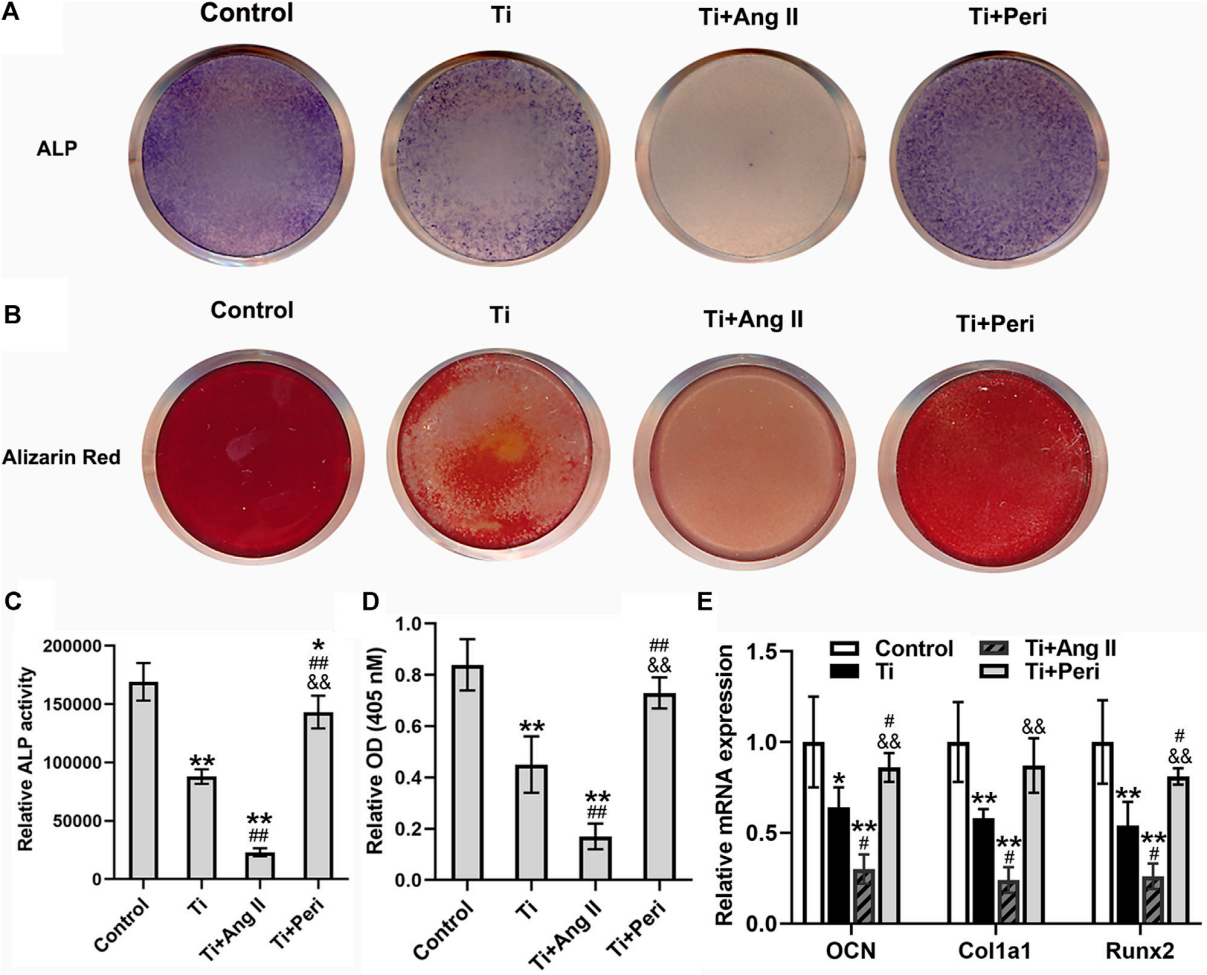
Ang II enhanced but perindopril repressed Ti-particle-induced suppression of osteogenic differentiation in mouse BMSCs. Data are representative images or expressed as the mean ± SD of each group from three separate experiments (*n* = 3 per group). **(A)** Representative images of ALP staining on day seven (photo). **(B)** Representative images of alizarin red staining on day 21 (photo). **(C)** ALP activity on day seven. **(D)** Mineralization quantitated from stained mineral deposits. **(E)** mRNA levels of osteogenic genes (OCN, Col1a1, and Runx2) on five. *p < 0.05 vs. the control group; **p < 0.01 vs. the control group; #*p* < 0.05 vs. the Ti group; ##*p* < 0.01 vs. the Ti group; &&*p* < 0.01 vs. the Ti + Ang II group.

The effect of Ang II or perindopril on Ti-particle-induced osteogenic differentiation in mouse BMSCs was examined by ALP staining and alizarin red staining. The results showed that Ti particles significantly decreased the staining density of ALP and inhibited ALP activity, and such effects were potently enhanced by Ang II but remarkably reversed by perindopril (Figures 6A,C). Moreover, alizarin red staining indicated that mineralization in cells decreased due to the stimulation of Ti particles, while even lower calcium mineralization was observed in the Ti + Ang II group, but these changes were abrogated by perindopril. (Figures 6B,D). Furthermore, RT-qPCR revealed that Ti particles apparently reduced the mRNA levels of osteoblast-specific genes, including OCN, Col1a1, and Runx2 (Figure 6E), and the levels were even lower under the treatment of Ang II but higher under the treatment of perindopril (Figure 6E). Collectively, these results demonstrated that Ang II enhanced but perindopril repressed the inhibition of osteogenic differentiation induced by Ti particles in mouse BMSCs.

### Inhibition of ACE Repressed Titanium-Particle-Induced Osteolysis by Suppressing the RANKL/RANKL Signaling Pathway and Activating the Wnt/β-Catenin Signaling Pathway

**FIGURE 7:**
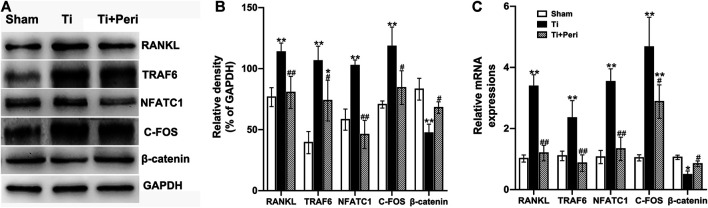
Inhibition of ACE repressed Ti-particle-induced osteolysis by suppressing the RANKL signaling pathway and activating the Wnt/β-catenin signaling pathway. Relative levels of RANKL, TRAF6, NFATC1, C-FOS, and β-catenin in mouse calvarial tissues to the control GAPDH expression were determined by Western blot and RT-qPCR. Data are representative images or expressed as the mean ± SD of each group from three separate experiments (*n* = 3 per group). **(A,B)** Western blot analyses. **(C)** RT-qPCR analyses. *p < 0.05 vs. the sham group; **p < 0.01 vs. the sham group; #*p* < 0.05 vs. the Ti group; ##*p* < 0.01 vs. the Ti group.

To further elucidate the potential molecular mechanism by which bone local RAS regulates Ti-particle-induced osteolysis, the mRNA and protein expressions of RANKL, TRAF6, NFATC1, C-FOS, and β-catenin in calvarial tissues were examined. Compared with the sham group, the Ti group presented significantly increased expressions of RANKL, TRAF6, NFATC1, and C-FOS and decreased expression of β-catenin. However, these changes were dramatically abrogated in the Ti + Peri group (Figures 7A–C), which indicated that inhibition of ACE by perindopril could suppress the expression of RANKL-mediated TRAF6, NFATC1 and C-FOS and promote β-catenin expression. Taken together, the results suggested that inhibition of ACE almost completely repressed the Ti-particle-induced osteolysis by suppressing the RANKL/RANK signaling pathway and activating the Wnt/β-catenin signaling pathway.

## Discussion

Studies have demonstrated that local bone RAS components are involved in many osteolytic diseases related to osteoclasts (Santos et al., 2015; Zhao et al., 2019), while osteoclasts play an indispensable role in the development of PPOL induced by wear particles (Jiang et al., 2013a; Jiang et al., 2013b; Gallo et al., 2013; Longhofer et al., 2017). One type of wear particles, Ti particles, has been reported to inhibit bone formation by suppressing osteogenic differentiation (Wang et al., 2016; Wang L. et al., 2020). Importantly, local bone RAS is found to participate in Ti-particle-induced inflammatory osteolysis, which may be a potential mechanism of pathogenesis in PPOL (Zhang et al., 2011). In line with these studies, the present study identified up-regulated expressions of three RAS components in interface membrane tissues from patients with PPOL and calvarial tissues of Ti-particle-induced osteolysis in mice, and these findings indicate the potential role of local bone RAS in PPOL. We further investigated the underlying mechanism of local bone RAS in wear-particle-induced PPOL by examining Ti-particle-induced promotion of bone resorption and suppression of bone formation using RAW264.7 macrophages and mouse BMSCs, and determined if inhibition of ACE by perindopril could suppress Ti-particle-induced osteolysis in a murine calvarial osteolysis model.

We examined the effect of local bone RAS on Ti-particle-induced osteolysis in a murine calvarial model. We observed a large area of bone erosion and a large number of bone resorption pores on the mouse calvarial surface in presence of Ti particles, which is in line with the findings in previous studies (Hu et al., 2017; Wang Q. et al., 2020). Whereas, inhibition of ACE by perindopril suppressed Ti-particle-induced osteolysis, as indicated by an increase in BMD and BV/TV, and a decrease in the number of pores and the percentage of total porosity. Moreover, an increase in the area of bone erosion with the increase of osteoclasts was present on the calvarial surface in the Ti group, which was inhibited by the treatment of perindopril, as illustrated by a decrease in the number of TRAP-positive multinucleated osteoclasts and the values of OCs/BS and ES/BS. These results are consistent with those of previous studies in the role of local bone RAS in osteoclast-related osteolytic diseases (Wang et al., 2018; Li J. et al., 2019; Akagi et al., 2020). Our results indicate that inhibition of ACE by perindopril plays a protective role in Ti-particle-induced osteolysis through inhibition of osteoclast formation and differentiation. In addition, Ti particles repressed bone formation and this effect was ameliorated by inhibiting ACE by perindopril. Furthermore, Ti particles increased the mRNA levels of osteoclast-specific genes and decreased those of osteoblast-related genes, while these effects, again, were abrogated by perindopril. Taken together, our study first reports that Ti particles stimulate bone resorption and suppress bone formation by up-regulating the expressions of RAS components, ultimately leading to osteolysis. Inhibition of ACE by perindopril exerts a potential therapeutic effect on wear-particle-induced PPOL.

The imbalance between osteoclastogenesis and osteoblastogenesis is known to be essential in wear-particle-stimulated PPOL (Jiang et al., 2013b; Gallo et al., 2013). Hence, we also investigated whether local bone RAS is involved in regulating Ti-particle-induced osteoclast formation, osteoclast-mediated bone resorption, and osteogenic differentiation. We found that Ti particles significantly increased the number and area of osteoclasts as well as osteoclast-mediated bone resorption, and such effects were enhanced by Ang II but inhibited by perindopril. In addition, Ti-particle-induced increases in the mRNA levels of osteoclast-related genes, such as TRAP, CK, and OSCAR, were significantly enhanced by Ang II but remarkably reduced by perindopril. Furthermore, Ti particles inhibited osteogenic differentiation, while this process was enhanced by Ang II but abrogated by perindopril. Meanwhile, Ti-particle-induced decreases in the mRNA levels of osteoblast-related genes, such as OCN, Col1a1, and Runx2, were enhanced by Ang II but suppressed by perindopril. Altogether, these findings indicate that Ang II enhances but perindopril suppresses Ti-particle-induced osteoclast formation as well as osteoclast-mediated bone resorption, and inhibition of osteogenic differentiation.

It is well known that the RANKL/RANK and Wnt/β-catenin signaling pathways function in wear-particle-induced PPOL by promoting bone resorption and inhibiting bone formation (Gohda et al., 2005; Nam et al., 2017; Park et al., 2017; Wang et al., 2021). RANKL is found expressed and upregulated in Ti-particle-induced osteolysis (Zhu et al., 2017; Li W. et al., 2019; Ihn et al., 2019), and it is a major reason for osteoclastogenesis and bone resorption in PPOL (Li W. et al., 2019). Combination of RANK with RANKL activates NF-κB signaling by recruiting adaptor molecules (such as TRAF6) and subsequently leads to the activation of C-FOS and NFATC1, which in turn up-regulates the expression of osteoclast-related genes and promotes the maturation and activation of osteoclasts (Gohda et al., 2005; Park et al., 2017). In this study, we found that the mRNA and protein expressions of RANKL, TRAF6, C-FOS, and NFATC1 in mouse calvarial tissues increased significantly with the presence of Ti particles, and such increase was evidently mitigated/reversed by the treatment of perindopril to inhibit ACE. Moreover, activation of the Wnt/β-catenin signaling is important to osteoblastogenesis and bone formation, and many studies have indicated that Ti particles inhibit bone formation by down-regulating the Wnt/β-catenin signaling pathway (Wang et al., 2016; Ping et al., 2017). In line with these exisiting findings, the present study revealed down-regulated mRNA and protein expressions of β-catenin with the treatment of Ti particles, while such effects were abrogated by perindopril. In short, the results here are consistent with those in our previous work that activation of local bone RAS increases bone resorption and decreases bone formation *via* regulating the RANKL/RANK/TRAF6 and Wnt/β-catenin signaling pathways (Wang et al., 2018). In this study, aberrant activation of RAS was found associated with up-regulated expression of RANKL and the downstream molecules of NF-κB pathway, including TRAF6, C-FOS, and NFATC1, as well as down-regulated expression of β-catenin in Ti-particle-induced osteolysis; whereas, these effects were alleviated by inhibition of ACE by perindopril. Therefore, we speculate that wear particles may activate the RANKL-RANK signaling pathway and inhibit Wnt/β-catenin signaling pathway through the mediation of local bone RAS, which induces bone resorption and suppresses bone formation, and finally leads to the occurrence of PPOL. This suggested that the RANKL-RANK and Wnt/β-catenin signaling pathways play an important role in the process of the Ti-particle-induced PPOL through the mediation of local bone RAS.

There are several limitations in the present study. Firstly, the wear particles that cause clinical PPOL are mainly polyethylene rather than Ti particles (Sun et al., 2020). However, it has been reported that both Ti and polyethylene can comparably imitate the authentic ability of wear particles to activate osteoclast formation and function to induce PPOL *in vivo* (Shao et al., 2015; Hu et al., 2017). Secondly, wear particles in patients are continuously released from the prosthesis at a low concentration (von Knoch et al., 2004), while in our experiments Ti particles were used in a fixed amount. Thirdly, we used a model of acute osteolysis induced by Ti particles, which is slightly different from the actual clinical situation. Fourthly, in patients undergoing joint replacement in clinic, the prosthesis is generally built into the long bone. Whereas, in the mouse model in this study, the flat skull with the periosteum removed was used as the medium for osteolysis, which lacked the loaded implants. Therefore, a mouse femoral Ti rod model would be more suitable for simulating osteolysis. Such a model will be evaluated in our future research. Finally, induction of inflammatory cytokines, such as tumor necrosis factor-α (TNF-α), interleukin-1β (IL-1β), and IL-6, by wear particles is critically important to the pathogenesis of PPOL and consequent aseptic loosening (Peng et al., 2015). The present study, however, did not explore the effect of local bone RAS on wear-particle-induced inflammatory cytokine production in the pathogenesis of PPOL. Research on this aspect is in need and will benefit our understanding of the pathogenesis of PPOL.

## Conclusion

In conclusion, our study shows that expressions of ACE, AT1R, and AT2R are up-regulated in the interface membrane from PPOL patients and calvarial tissue from Ti-particle-induced mouse osteolysis model, indicating that aberrant activation of local bone RAS contributes to PPOL induced by wear particles. Moreover, Ang II enhances but perindopril inhibits Ti-particle-induced osteoclast formation and osteoclast-mediated bone resorption, as well as the suppression of osteogenic differentiation *in vitro*. Activation of RAS increases RANKL expression to activate the RANKL/RANK signaling and decreases β-Catenin expression to inhibit the Wnt/β-Catenin signaling *in vivo*. As a whole, our findings contribute to understanding the molecular mechanism of the pathogenic process of wear-particle-induced PPOL after TJA operation, and such understanding may shed light on the potential preventive or therapeutic treatment for PPOL.

## Data Availability

The original contributions presented in the study are included in the article/Supplementary Material, further inquiries can be directed to the corresponding author.
